# Tau is required for pneumonia‐induced cerebrovascular dysfunction

**DOI:** 10.1002/alz.091920

**Published:** 2025-01-03

**Authors:** Samantha D Chaney, Lauren H McAdams, Meredith S Gwin, Troy T Stevens, Mike Lin, Amy R. Nelson

**Affiliations:** ^1^ University of South Alabama, Mobile, AL USA

## Abstract

**Background:**

Many survivors of lung injury, including those with bacterial pneumonia and COVID‐19, suffer from incident dementia. Patients who have had pneumonia and other infections are at a higher risk for developing Alzheimer’s disease and related dementias (ADRD) (Chu et al., BBI, 2022, Sipila et al., Lancet Infec Dis, 2021, Girard et al., J Gen Intern Med, 2018). Previous experimental studies have shown that tau is required for pneumonia‐elicited deficits in long‐term potentiation (Balczon et al., FASEB J, 2021). Neurovascular unit dysfunction, including breakdown of the blood‐brain barrier (BBB) and gliosis, occurs early in the ADRD pathophysiological cascade. We have shown that bacterial pneumonia causes BBB breakdown, gliosis, and increased tau phosphorylation in the brain. Here, we investigated whether tau is required for pneumonia‐induced cerebrovascular dysfunction.

**Method:**

C57BL/6J or tau knockout (KO) adult mice (1‐3 months old, both male and female) were intratracheally infected with *P. aeruginosa* (PA103, ExoU and ExoT competent, 10^5^ CFU) for 24 hours. Next, we performed immunofluorescence staining for CD13 (pericytes), lectin (endothelial cells), IgG (BBB leakage), GFAP (reactive astrocytes), and Iba1 (microglia) on serially sectioned brain slices, and imaged and analyzed hippocampus and cortex.

**Result:**

BBB leakage and reactive astrocytes and microglia were increased in the hippocampus and cortex 24 hours post‐infection with PA103 compared to saline in C57BL/6J mice. Pericyte coverage of capillaries was reduced in the hippocampus 24 hours post‐infection with PA103 compared to saline in C57BL/6J mice. There was no significant difference in BBB leakage, pericyte coverage or gliosis 24 hours post‐infection with PA103 compared to saline in tau KO mice.

**Conclusion:**

Tau is required for bacterial pneumonia‐induced BBB breakdown and gliosis, revealing a novel immune role for this pathological hallmark of ADRD. Future studies should focus on 1) mechanism(s) by which pneumonia‐induced tau causes neurovascular unit dysfunction, 2) whether cytotoxic tau produced by the lungs during infection contributes to ADRD, 3) how the tau generated in pneumonia compares to ADRD tau variants and phosphorylation states, and 4) the applicability and benefit of therapeutics targeting tau, such as anti‐tau antibodies, on incident dementia associated with pneumonia and/or on cerebrovascular dysfunction in mild‐cognitive impairment and ADRD.

